# Hijab No More: A Phenomenological Study

**DOI:** 10.1007/s10943-020-01068-7

**Published:** 2020-07-23

**Authors:** Jhanghiz Syahrivar

**Affiliations:** 1 Institute of Marketing, Corvinus University of Budapest, Fővám tér 8, Budapest, 1093 Hungary; 2 School of Business, President University, Jl. Ki Hajar Dewantara, Bekasi, 17530 Indonesia

**Keywords:** Hijab dissociation, Compensatory consumption, Muslim minority

## Abstract

The consumption of Islamic products has been discussed quite extensively in many studies, yet the opposite case which is the dissociation from Islamic products is rarely discussed. This study aims to investigate the phenomena in which Muslim women who were raised with conservative values at home countries choose to dissociate from hijab when they live in the western countries. Moreover, they become activists who discourage other Muslim women from wearing hijab. This study adopts a phenomenological research design. The results suggest that hijab dissociation is a form of compensatory mechanism aimed at minimizing self-discrepancy, restoring self-esteem, gaining personal control, reducing perceived alienation and coping with psychological trauma. This study contributes to the theoretical gap in compensatory consumption literature by linking the theory with the non-consumption of religious products.

## Introduction

In the modern time, veiling in public space can be political matters (Sinclair 2012; Al-Mahadin 2013; Rosenberg 2019). It is a case where the woman’s body becomes a political arena, torn apart between secular versus religious values. Muslim jurists and scholars are somewhat divided as to whether wearing a hijab (a piece of cloth on one’s head) for Muslim women is compulsory or optional (Ahmed 2020). Nevertheless, the concept of “hijab” as “modesty” for Muslim women seems to be agreed upon (Hwang and Kim 2020). The Muslim jurists and scholars are only in disagreement about which parts (of the body) to cover which explains the varieties of designs (e.g., styles and lengths) of hijabs in Muslim-majority countries.

The verse concerning hijab is derived from the Qur’an, Surah al-Noor Chapter 24 Verse 31:

And tell the believing women to reduce [some] of their vision and guard their private parts and not expose their adornment except that which [necessarily] appears thereof and to wrap [a portion of] their head covers over their chests and not expose their adornment except to their husbands, their fathers, their husbands’ fathers, their sons, their husbands’ sons, their brothers, their brothers’ sons, their sisters’ sons, their women, that which their right hands possess, or those male attendants having no physical desire, or children who are not yet aware of the private aspects of women. And let them not stamp their feet to make known what they conceal of their adornment. And turn to Allah in repentance, all of you, O believers, that you might succeed.—Sahih International (Quran 2020).

In the recent years, there have been some interesting phenomena in which Muslim women, especially those from countries with conservative Islamic values, go online to express their attitudes about compulsory hijab (Seddighi and Tafakori 2016). For instance, My Stealthy Freedom movement was initiated by Masih Alinejad, an Iranian woman and a political activist, in 2014 against compulsory hijab in Iran. Her movement has since become a global phenomenon and attracted not only Muslim women who are against compulsory hijab but also Muslims or ex-Muslims who are against hijab in general, whether it is compulsory or optional. The latter typically live in the West where they have greater freedoms to express their attitudes about Islam through social media platforms, such as Facebook, Twitter and Youtube.

A study by Fani et al. (2020) in the context of Iranian women provides fresh evidence of hijab dissociation among Muslim women. Their study emphasizes on the role of beliefs in the adoption/rejection of hijab. This study, however, takes a different angle by examining Muslim women in the non-Muslim-majority country and investigating factors that go beyond one’s religiosity or beliefs about Islam or Islamic values. Moreover, this study has a special interest in not only women who stop wearing hijab in the Western countries but also those who actively promote hijab dissociation.

In customer behavior study, dissociation occurs when customers attempt to separate themselves from certain products or brands because they are conflicting to their new-found identities. Mandel et al. (2017) in their compensatory consumption model include dissociation as a form of compensatory mechanism against self-discrepancy. Apart from the declining of faith (Fani et al. 2020), previous studies have also revealed that Muslim women might distance themselves from their cultures and Islamic identities in order to mitigate social barriers with the locals and as a result of conflicting or new-found identities in the secular environments (Weber 2016; Mohammadi 2018; Finn et al. 2018). While studies centered on hijab adoption are exhaustive, the reverse case which is dissociating from hijab is rare.

The research questions are multifold: 1) What are the antecedents of hijab dissociation? 2) What are the consequences of dissociation in hijab context? 3) Is hijab dissociation a form of compensatory mechanism?

The rest of this paper is presented as follows: First is the literature review section where the core theory of this study is discussed. Second is the methodology section where research steps taken in this study are elaborated. Third is the findings section where the results of the interviews are discussed and explained. Fourth is the discussion section where general relationships between antecedents and compensatory consumption are discussed and the nature of dissociation is elaborated. Fifth is the conclusion section where the summary, recommendations for future studies and managerial implications are offered.

## Literature Review

According to Woodruffe and Elliott (2005), compensatory consumption occurs when there is a disconnection between the need and the actual purchase. Therefore, certain consumptions are essentially meant to compensate one’s disappointment for being unable to obtain desired products. Moreover, compensatory consumption also occurs as a response toward negative psychological conditions or emotional states due to the gap between actual versus ideal-self (Jaiswal and Gupta 2015; Mandel et al. 2017).

Consumers reduce uncertainties and preserve their self-concepts by engaging in compensatory consumption (Woodruffe-Burton and Elliott 2005). Consequently, as suggested by Kim and Rucker (2012), compensatory consumption is not exclusive to self-enhancement consumptions but also encompasses self-verification (or affirmation) consumptions in order to project one’s self-concept more accurately (instead of positively). For instance, the study conducted by Brannon (2016) suggests that consumers with negative self-view would gravitate toward products which signal or verify their negative self-view. Compensatory consumption is further comprised of a wide range of—sometimes chronic and neurotic—consumption behaviors, such as conspicuous consumption, retail therapy, compulsive buying, addictive consumption, self-gift giving, impulsive buying and compensatory eating (Woodruffe 1997; Kang and Johnson 2011; Koles et al. 2018).

Consumers from low-income hence lack of power may engage in compensatory consumption by purchasing high-status products in order to repair their ego as well as to shield themselves from future threats to self (Rucker and Galinsky 2008; Sivanathan and Pettit 2010; Jaiswal and Gupta 2015). Purchasing high-status or power-related products are not the only mode of compensatory consumption. In the expanded model of conspicuous consumption, customers may buy products that are rare in circulation to project their unique self (Gierl and Huettl 2010). Alternatively, inferior and counterfeit products can be sought after by youngsters in order to instill envy and avoid social exclusion (Abdalla and Zambaldi 2016).

Previous studies on compensatory consumption linked the concept with gender studies. For instance, Woodruffe (1997) links compensatory consumption with feminism, the idea that shopping not only helps women regulate their emotions but also empowers them because it gives them a sense of personal control in their households. In the subsequent studies, compensatory consumption is also linked to manliness (Woodruffe‐Burton 1998; Holt and Thompson 2004; Chiou et al. 2013; Witkowski 2020). For instance, a man whose concept of masculinity is threatened would gravitate toward products or activities that symbolize manliness (e.g., leather jacket, sport cars and manly scents cologne).

Mandel et al. (2017) theorize five compensatory mechanisms: addressing directly the source of self-discrepancy, symbolically signal one’s mastery in area of self-discrepancy, distracting oneself from the self-discrepancy, compensating in other area that is unrelated to the area of self-discrepancy and dissociating oneself from the source of self-discrepancy. This study places a great emphasis on dissociation, a less discussed compensatory mechanism.

According to Dunn et al. (2013), dissociation is…

The act of avoiding and disparaging products and brands that represent undesired groups or identities. (p. 274)

There is a link between traumatic experience and dissociation. A study by Spitzer et al. (2006) provides a brief history of dissociation theory and its developments. They argue that dissociation is a part of personality disorder which stems from traumatic experiences. Meanwhile, Cardeña (1994) argues that dissociation is a form of defense mechanism with the aim of safeguarding one’s psychological integrity and protecting oneself from external stimuli that can trigger distress or remind a person of his or her traumatic experiences. Moreover, in an empirical study by Stolovy et al. (2015) involving 150 women proves that traumatic history (e.g., childhood trauma) is a predictor of dissociation.

One of the features of dissociation is detachment from objects that induce stress or evoke traumatic memories (Schillaci et al. 2009). According to Zittoun (2009), an object may become symbolic because it is linked to past events, which could be pleasant or unpleasant, especially concerning one’s interactions with significant others in the past. A study by Wang (2013) highlights the relationship between emotion and memory and suggests that women are better than men in recalling past negative events. In this respect, hijab as an object of disputes among Westerners who question its meanings as well as motives (Sinclair 2012; Al-Mahadin 2013; Rosenberg 2019) can induce negative emotions or evoke unpleasant memories among Muslim women who live in the West hence dissociation behavior.

Previous studies suggest that Muslim women dissociate from their cultures and Islamic identities (e.g., hijab) in order to avoid social discriminations and to make coherent with their new-found identities (e.g., feminist) in the Western countries (Weber 2016; Mohammadi 2018; Finn et al. 2018; Fani et al. 2020). According to White et al. (2012), when an aspect of social identity is threatened, those higher in independence demonstrate a dissociative response to identity-linked products. In this respect, the dissociative response is driven by self-worth concerns.

Globalization (and Westernization) may change people’s attitudes toward their religions. For instance, a study by Maliepaard et al. (2010) suggests a declining religious attachment as well as religious practices among second-generation Muslim minorities who live in the Netherlands. Ideally, religions (religious resources) can promote well-being (Van Cappellen et al. 2016). However, those who are unable to cope with their stress and trauma through religious or spiritual means are more likely to dissociate from them (Ren et al. 2019).

## Methodology

This study is a qualitative analysis following an inductive approach. I employed phenomenological research design as outlined by Groenewald (2004). My epistemological position regarding this research can be formulated as follows: a) Data are contained within the perspectives of Muslim women who are knowledgeable and are subjects to a phenomenon under investigation (e.g., hijab dissociation phenomenon), and therefore, b) I as the researcher engaged with the participants in collecting the data (e.g., by gathering their perspectives) in order to unravel the phenomenon.

As advised by Groenewald (2004), I used a purposive sampling technique by relying on my judgement and the purpose of the research. First, I determined the criteria of my informant: 1) a Muslim (or ex) woman who currently lives in the West (e.g., Europe, the USA) where Muslims are a minority group, 2) she wore hijab regularly before immigrating to the Western country, 3) she decides to remove her hijab (dissociation) at some point after living in the Western country, and 4) she is an advocate/activist who discourages Muslim women from wearing hijab. The four criteria were chosen to capture the phenomenon under investigation which was hijab dissociation as well as its promotion among Muslim women in the Western world.

Second, I looked on social media platforms (e.g., twitter) where people occasionally shared their thoughts about certain issues. I used hashtag such as #FreeFromHijab, #MyStealthyFreedom and #NoHijabDay, and I found some female twitter users sharing their thoughts about hijab dissociation phenomena.

Third, I contacted them via a private message feature in a social media platform and asked them to discuss further over emails or video calls. In total, there were 26 participants or informants for this study but in the end I thought there was only one informant who satisfied all the criteria mentioned above, captured the phenomenon under investigation and agreed for a series of in-depth interviews. The remaining 25 informants partially satisfied the criteria (e.g., remove their hijabs while living in the West but unwilling to be called advocates or activists of hijab dissociation), and their stand points on hijab would be used to improve the validity of this study. The main informant in this study is a Lebanese (ex) Muslim immigrant who currently lives in Quebec, Canada. By the time this article is written, she is 24 years old, married and an active campaigner of no hijab. I came to know her via twitter, a social media platform where she regularly expressed her anxiety about Islam, Muslim and the symbols associated with the religion (e.g., hijab). The hijab dissociation phenomenon in this study was explored based on her unique point of view. I would like to highlight that although the insights provided in this study is mainly based on one account, but I had engaged with the informant for half a year through a series of online interviews (e.g., video calls) and email exchanges, giving me the opportunities to dig deeper and judge the informant’s consistency and accuracy. According to Hycner (1985), phenomenological research focuses on qualitative issues instead of quantitative ones given the vast amount of data generated from one interview only. The research comes to an end when saturation level is achieved, meaning that no new emerging themes can be generated from the interviews.

Fourth, I established honesty and confidentiality as outlined by Groenewald (2004) so that the data gathering went smoothly. This was achieved by sending a written statement on the purpose of the research, my expectations as a researcher and also a clause mentioning the confidentiality or animosity of the research participant in this research. The participation in this research is voluntary, meaning that no money involved in this research but I have promised the research participant to send her the article once it is published.

Fifth, I began a series of semi-structured interviews through various methods, such as email exchanges where the informant was asked to write a narrative regarding her experience with hijab, social media and video calls and by bearing in minds that convenience, comfort and security are the top priorities for the informant. A combination of video calls and email exchanges is typically sufficient to capture the verbal and non-verbal messages.

Sixth, text coding and decoding and important themes were inferred from the codes (inductive). In total, there were 4567 codes which were analyzed for this study and a total of five themes were generated.

Seventh, I conducted validity checking by returning the results to the informant in order to determine if the essence of the interview had been fully captured. Also the responses given by the informant were always compared against previous studies, hence allowing me to reflect on each response and figuring out if there was any similarity or contradictions. To further improve the validity, I also conducted a survey about hijab adoption involving 25 Muslim participants who live in Europe. The participants partially satisfied the four criteria as mentioned in the first step.

Table 1 encompassed interview questions aimed at answering the central research questions explained in the introduction.

**Table 1:** Core interview questions

No.	Section	Items
1.	Religiosity and attitude	1. How religious are you? Please compare your religiosity with your parents, friends and the society that you live in!
		2. In general, what is your attitude toward Islam and Muslims?
		3. In general, what is your attitude toward religions in general and religious people?
2.	Self-identity/Self-concept	1. As a person, how do you describe yourself? What is your self-concept?
		2. Is there any particular group (e.g., political or non-political groups) that you identify yourself with? Are the members of this group mostly Muslims or non-Muslims?
3.	Hijab Symbolism and Dissociation Motives	1. Have you experienced a negative event while living overseas because people are aware that you are a Muslim?
		2. In general, what are the attitudes of the people who are close to you about Islam and Muslims?
		3. When did you start wearing hijab and when did you stop wearing it?
		4. What image or symbol does hijab represent to you? Alternatively, what comes to your mind when you look at hijab?
		5. How is your family respond after you stop wearing hijab? What about your friends who are close to you?
4.	Campaigning Free Hijab	1. When did you start campaigning free from hijab? How? Why?
		2. Are there more women like you who stop wearing hijab? Are you close with them? Do you admire them?
		3. Do they actively promote free from hijab to other Muslim women?
		4. Are there people around you who are non-Muslims who actively promote freedom from hijab?
5.	Self-worth	1. How do you feel after no longer wearing a hijab?

### Findings

I presented several noteworthy codes and the themes (cluster) in Table 2. The themes generated in this study are 1) Religious Discrepancy, 2) Low Self-esteem, 3) Powerlessness, 4) Alienation and 5) Trauma.

**Table 2:** Themes and codes

Themes	Noteworthy codes
Religious discrepancy	Not religious, despise, atheist, dangerous, violence, hate, intolerance, fairy tales, *haram* (forbidden), stop believing, creation of men, cannot understand, problematic, does not make sense, [to] question, no proof, [to] criticize, toxic, …
Low Self-esteem	Less beautiful, less desirable, look darker, self-hate, obsessed, scars, uncomfortable, low self-esteem, depression, have nothing, struggle, not positive, pessimistic, feeling down, feeling trashy, suicidal, is not loved, bad person, bad intention, belittled,…
Powerlessness	No independence, no freedom, secret, pressure, burden, prison, oppressive, control, honor, fabrication, power, symbol, brainwash, restricted mobility and choice,…
Alienation	Lost, disowned, abandoned, shut down, being avoided, [to] distance, being an alien, disconnected, …
Trauma	Beaten, bullied, hit, abused, insulted, put one’s down, mistreated, abuse of power, sadistic,…

#### Religious Discrepancy

The first thing I inquired from the informant was about her religiosity as reflected in her attitude about Islam. Religiosity is central to ethical or moral judgement (Ward and King 2018) as well as consumer decision-making (Islam and Chandrasekaran 2020), such as whether to purchase or not to purchase hijab. In this regard, Religious Discrepancy is a theme which represents responses and narratives where the informant claimed to lose her faith in Islam leading to her decision to dissociate herself from hijab. The following excerpt from the in-depth interview illustrates her attitude about Islam after living in Canada:

“After many researches and personal experiences, I now despise Islam… To me, Islam is a dangerous tool that condones violence and encourages hate and intolerance… I stopped believing in the God of Islam because He had human characteristics. I knew it was a creation of men so the Qur’an was also creation of men. I saw how Mo [Muhammad] was abusing His power, manipulative and hated the image of the “real” good muslimah woman that deserved respect. Apparently we don’t live in this world for our own desires but for Sadistic God’s pleasure. Torture, execution and stoning of infidels, apostates, gays is like a show for Sadistic God who finds pleasure in watching people suffer and dying in His name.”

Notice the statement “I now despise Islam” which suggests that the informant did not have this attitude before or prior to living in Canada. Her attitude toward Islam also extends to religions in general:

“I later started making researches about him [Muhammad], his life, and made sure not to read informations about him in anti-Muslim websites. I later completely stopped believing in any gods. I believe that if there is a very powerful energy in this world that created everything, that we should be able to detect that energy in any way. If God is so extraordinarily powerful, we should be able to detect him with science but we don’t. No energy. No trace of him nowhere. No proof. Nothing. So this is the story of how I became an atheist.”

The informant mentioned the word “science” as a justification of her attitude. The previous study by Ren et al. (2019) suggests that people who are highly educated tend to deviate from religious or traditional values.

The informant explained what hijab means to her after she left Islam:

“I don’t believe hijab is a tool of oppression but a tool fabricated by the Islamists [Islamic political movements/actors] in the mid 20th century as a response to the massive unveiling of women during and after western imperialism, by creating a new type of veil, the one that we know today, meant to control society… because women are believed to be their family’s honor and by extension, their society’s honor, Islamists knew they were losing power and blamed wars and catastrophes on general immorality of the people… And because women are the society’s honor they must be controlled so that the Islamists can control the people… The veil became tightly associated with Islam, it became its symbol… So the Islamists have to symbolically control the people by making women wear their newly created version of hijab… Take hijab as a signature of this new Islam… I believe this is the reason why they make campaigns to veil young girls and scare children with hell and djinns [supernatural entities] only so that they can have that control even later in their life because they know it’s easier to brainwash and veil a child than a mature adult.”

The above statement also supports Fani et al. (2020) who argue that the dissociation from hijab is a consequence of the declining importance of Islamic values in the individuals. However, it is important to note that in the case of the informant in this study, it is not the sole reason.

#### Low Self-Esteem

Low Self-Esteem is a theme which represents responses where the informant claimed to have lost her sense of self-worth. The following excerpt from the in-depth interview illustrates her outlook about life in general:

“I still struggle in my everyday life because I was so brainwashed into thinking this life is worthless and that all that mattered was life after death. I can’t always see the positive in life and things in general and am quite pessimistic.”

A study by Heinonen et al. (2005) among Finland participants demonstrates that low self-esteem is a predictor of pessimism in adulthood. The fact that the informant has a negative worldview which is an indicator of her low self-esteem.

The informant felt that she was less desirable as a woman and that she was convinced by her family members that wearing hijab was the solution:

“I was told that I was less beautiful and desirable than my sisters because I’m naturally tan. I was told what colours to wear and not to wear because they’d make me look darker. I was told I looked like a tree when I wore green! That I shouldn’t wear red, black, white, yellow…”

“I still feel uncomfortable sometimes as I have low self esteem and was told by family members that I wasn’t beautiful without a hijab.”

Wilhelm et al. (2018) argue that Muslim women’s perceptions on their body image were affected by their veiling. Surprisingly, they found that veiled Muslim would feel more attractive when they were in the state of being unveiled. Indeed, the informant claimed that she was more attractive and more confident when not wearing a hijab. She further elaborated her childhood experience:

“I was taught to hate myself for what I looked like because I wasn’t pretty enough or white enough. I was obsessed with trying to look white with make up and dyed my hair lighter. I was never taught to love my looks, my natural hair, my natural colour. I grew up hating myself and my looks. Now I am left with the scars of the past and trying to build up my confidence from scratch. I’m not crying when looking at myself in the mirror. I’m not trying to change my appearance to please others. This is simply me, and I like it.”

The above statements support the study by Zine (2001) among Muslim youth in Canada, a predominantly white society, where women with dark complexion are perceived less beautiful. Therefore, Muslim women “of color” would derive their sense of (inner) beauty and, more importantly, respect from their peers by wearing hijab (Droogsma 2007).

Rosenberg (1962) suggests that religious dissonance could result in lower self-esteem. When a person adopts a religious view which is in conflict with the society he or she lives in, it would promote anxiety, tension and emotional distress, hence a negative evaluation toward the self. I assume that Rosenberg’s explanation partially explains the low self-esteem of the informant.

#### Powerlessness

Powerlessness is a theme which represents responses and narratives where the informant expressed her feeling of being powerless, lack of freedom and independence leading to her decision to dissociate herself from hijab. The following response suggests how hijab was perceived by the informant as a “prison” or an antithesis of freedom:

“I think about pressure, a burden because I always felt like I had to represent Islam and was always associated to it even when I stopped believing in it and was forced to wear it. When I think about the hijab I imagine a prison and I remember how uncomfortable I was when I used to wear it. It really is to me an oppressive tool.”

The above statement provided by the informant supports Jackson and Monk-Turner (2015) who found that 21% of their samples (Egyptian women) equated hijab with “prison” which symbolizes the suppression of freedom.

The informant claimed that the fact that she was forbidden to remove her hijab in public by her family members was an indicator that she did not have the (freedom of) choice to begin with. A much detail narrative was retold by the informant as follows:

“I always wanted my independence but could never stand up to my mother because I was scared of her. I’m a university student so I need to stay on campus some days til late at night, especially when I have exams. My mother would call me non stop, always interrupt my studies and would even come to my uni to pick me up like I was a 10 y/o child. I’m a woman in my mid twenties and I need my space. So my mother’s behavior was too much for me. One morning she followed me when I left to go to uni by bus and saw me removing my hijab. I used to wear it when I’d get out the front door of the house and remove it at the corner of the street. My mom says she saw me removing it at the subway station and that I was with a blond man [giggle] I always removed my hijab at the corner of the street before my bus stop. Anyway she became a nightmare after that and I didn’t know why because she never told me she followed and saw me removing the hijab. At that time I was already bringing stuff to uni and putting them in my locker. I was slowly moving out. One day while I was out with one of my sisters, my youngest sis texted me crying and angry. My mom had been yelling at her and telling her she was becoming “a bitch” like me. I was really shocked to hear that. So that night I decided that was it I was leaving. I wasn’t going to let my mom say such things and bully me and now she was doing it to my lil sister. So I decided to bring more and more stuff to uni. One evening right when I went to bed, my mother decided to search in my school bag because we had no privacy and noticed it was filled with clothes and my personal belongings. I woke up to her emptying my bag and I was terrified I had been discovered. My life was over. After a horrible evening and sleepless night, I told my mother in the morning that I was leaving. She didn’t want to let me go but with the help of my sisters I was able to pack my bags and left the house scared crying and shaking. I think it was the scariest day of my life but I’m proud I left at that time because I was told that night that my mother knew about me removing the hijab. I knew I was going to lose all the freedom I worked so hard to get. So I just left and here I am one year later being my best self.”

The above narrative was somewhat contradictory to the claim among Muslim women that hijab was a symbol of freedom and empowerment (Ruby 2006; Droogsma 2007; Carland 2011). Indeed, whether hijab is a symbol of freedom or an antithesis of freedom has been a long debate and a subject of discursive research (Hussein 2007). A study by Wayland (1997) on hijab in France suggests that although wearing hijab was seen as a manifestation of religious freedom but at the same time it could infringe the freedom of others when wearing hijab constitutes:

“…acts of pressure, provocation, proselytizing, or propaganda; undermine the dignity or the liberty of the student or of other members of the educational community; compromise their health or their safety; disrupt the conduct of teaching activities and the instructional role of teachers; and lastly disturb order in the school or the normal functioning of public service.” (pg. 553)

#### Alienation

Alienation is a theme which represents responses and narratives where the informant felt that she was being alienated by Muslims and disowned by her significant others due to her attitude about Islam and her decision to dissociate herself from Islamic identities (e.g., hijab). The following response suggests how removing hijab in public can lead to severe consequences, such as being disowned by family members:

“I want everyone to know I’ve lost everything because I wanted my freedom. I want everyone to know I was disowned by my parents because I decided to remove the hijab and live in uni residences instead of doing the 1 h 40 min ride every morning and every night. I want everyone to know my mother prays for my death everyday. That I was called a “sharmouta” [slut] for removing the hijab. That I have no more family and that they abandoned me after they messed up with my head for 23 years.”

Leaving Islam also means losing some old friends:

“In 2016, about a year after I rejected islam, I came across an old very close friend of mine. It was the first time she saw me without a hijab but told me she was ok with it, that it was my choice to wear it or not. We talked about different things, what we became after high school and other crazy stuff we’ve done and I talked about the day I tried alcohol. She told me she also occasionally drinks alcohol and smokes weed. I was surprised but happy to know she was open minded. I later told her honestly that I actually left Islam because I just stopped believing in God and that things started to make no sense to me. She was shocked. We chatted for a bit after that and shared numbers and she told me she had to go home. At that moment I was so happy to have seen her after all those years and thought she was so open about the choices I’ve made. But then I would text her, try to meet with her again and she’d always say she was busy. Then slowly she completely stopped answering my messages. And finally she blocked my number. I think she wasn’t ready to hear me say I left Islam. She was fine with me drinking, seeing a guy and removing the hijab but not leaving her dear and precious religion. How hypocritical is that?”

A study by LeCount (2017) affirms that “losing friends” upon announcing one’s exit from his or her religion is among the factors feared by the apostates. For this reason, ex-Muslims can choose to simply being silent on their lack of beliefs about Islam while abandoning Islamic practices in secret. A term “Cultural Muslim” applies to non-practicing Muslims yet still retain their Islamic identity (Milani 2017). According to Milani (2017), Cultural Muslims represent the “silent majority” and they are growing in numbers, especially in the West.

A Muslim can also face social alienation and discrimination in a non-Muslim-majority country. The informant told me how she struggled when she still wore a hijab in public spaces:

“I was called a taliban in Toronto and that I should “go back to my country” I’m a Lebanese Canadian… You feel like an alien because of your Muslim only upbringing.”

The above testimony supports Finn et al. (2018) conclude that Canadian Arab youth attempt to mitigate social discrimination by dissociating themselves from their Arab identity and by extension, Islamic identities (e.g., hijab).

#### Trauma

Trauma is a theme which represents responses and narratives where the participant claims to be subjected to a series of mental and physical abuses. The previous study by FitzPatrick et al. (2019) centered on victims of domestic violence suggests that compensatory consumption (e.g., dissociation) is used to cope with psychological trauma. The following response suggests the informant was aware that her attitude toward Islamic practices (e.g., wearing hijab) had dire consequences:

“Because I was beaten and bullied, I can’t help but feel fear when I meet muslims. But I work with muslim people that I got to know very well so I only have to watch what I say when they are around. Also, because I used to be a muslim and know that I have never been a bad person with extreme ideologies, I know most Muslim people surrounding me aren’t bad. I just prefer to distance myself from them.”

A study by El-Abani et al. (2020) on Libyan immigrants in the UK suggests that Muslim women who fail to observe Islamic duties, such as wearing hijab, is regarded as bringing dishonor upon their households, and hence, family members (e.g., husbands) may exact punishments to such women, such as by beating them. According to Spitzer et al. (2006), dissociation can occur as a result of traumatic experience. One feature of dissociation is detachment where patients distance themselves from the source of stress or trauma. In this regard, the informant perceived hijab as an object that induced her traumatic experience.

The parent–child relationship, especially mother–child interaction, can be the source of traumatic childhood experience:

“I was also mentally abused. I was when I was a child from my mother that I have a problem in my brain, that I’m handicapped. She said that about me to my sisters. She would constantly put me down and treat me like trash. I was suicidal at age 11, I thought my mother would be happier if I died because she would tell me she regrets giving birth to me, that I was the reason her life is so complicated and made me clearly understand that I wasn’t loved. But then she would say I am the one who doesn’t love her because I am a bad person with bad intentions.”

Hammond et al. (2000) argue that mothers with negative parenting histories exert more control in the lives of their children as a way to reduce their own anxiety level. Moreover, they have difficulties in allowing their children to have more independence due to negative treatments they received in their childhoods. The informant narrated the occasion in which her mother pried on her privacy:

“My mother knew about my secret [taking off her hijab in public] when a woman she knows saw me in the subway [without hijab] and told some members of my family about it. My mother then followed me one morning when I was going to university. She didn’t tell me about it at first and was trying to act like everything was normal but I noticed she became even more annoying, as trying to steal my phone at night and look into my school bag.”

The informant perceives Hijab as a symbol of control on Muslim women and their honor. Thus, it is not difficult to imagine when the informant sees her mother wears hijab (or any Muslim women), it would remind her of this authoritative symbol as well as her painful memories. She further argued on the motive of hijab:

“They have to keep the society under control and women are meant to hold that symbol [hijab] because they’re the society’s honor. I hope this makes sense.”

The manifestation of Islamic conservative values, aside from compulsory hijab for Muslim women, is the encouragement or persuasion to fight in the name of God and die as a martyr. The following excerpt from the in-depth interview suggests that the informant was raised in a Muslim family that adopted such values:

“My mother once told me that if I were a boy she would have sent me to Syria to fight with the Hezbollah [Islamic militant group] whether I’d like it or not because it’s an honor to fight in the name of Allah and also because it is an honor for a parent to have its child die as a shahid [a martyr]”

Previous studies have suggested a link between religious extremism, patriarchal society and domestic violence among Muslim women (Ghosh et al. 2017; Imran et al. 2020; Jamil 2020).

#### Hijab and Choice

Many Muslim women would claim that decision to wear hijab is a personal choice (Franks 2000; Afshar 2008) which is contradictory to the testimony provided by the informant in this study. She insisted that the idea that hijab was a personal choice was no more than public relations statements. According to her, in reality, many Muslim women did not have the choice; they were simply made to believe so. To improve the validity, I also surveyed 25 Muslim women who live in Europe through personal network and snowball sampling (Table 3). In brief, the participants were Muslim immigrants who came from Azerbaijan (4), Iran (3), Indonesia (7), Turkey (4), Egypt (2) and Pakistan (5). They were somewhere between 25 and 35 years old with education backgrounds from Masters (19) to PhDs (6). To be noted, these 25 Muslim women partially satisfied the criteria mentioned in Methodology section. The survey aimed to explore to what extent the adoption of hijab was a personal choice among Muslim participants in order to further strengthen the results of this study.

**Table 3:** Hijab and personal choice

No	Items	Results	Note
1.	Wearing hijab is a personal choice	As high as 96% of participants believe that wearing hijab is a personal choice	Notice that questions no. 1, 2 and 3 are basically probing the extent at which hijab is a personal choice. Responses no. 2 and 3 suggest that wearing hijab is not truly a choice as many Muslim women claimed it was
2.	I choose to wear hijab out of my own free will	However, only 76% of participants claimed to wear hijab out of their own free will	
3.	Women are completely free to choose to wear or not to wear hijab	And only 52% of participants believed that they were free to choose to wear or not to wear hijab	
4.	I have been introduced to hijab since my childhood	56% of participants said yes	Questions no. 4, 5, 6, 7, 8 and 9 are probing about the influence of parents on hijab adoption. The responses suggest that their parents do influence their decisions to adopt hijab to some degree
5.	I have adopted hijab for a long time	88% of participants said yes	
6.	My mother wears a hijab in public	96% of participants said yes	
7.	My parents remind me to wear a hijab	36% of participants said yes	
8.	My parents instruct me to wear a hijab	32% of participants said yes	
9.	My parents show favorable attitudes toward a daughter who wears a hijab	44% of participants said yes	
10.	My female friends or circles mostly wear hijab	64% of participants said yes	Questions no. 9 and 10 are probing about the possible influence of participants’ female circles on their decisions to adopt hijab. The responses suggest that most participants are surrounded by women who also adopt hijab
11.	Most women in the community where I mostly spend my life wear hijab	72% of participants said yes	
12.	Most men in my community favor women who wear hijab	56% of participants said yes	Questions no. 11 and 12 are probing about the possible influence of the opposite gender on the participants’ decisions to adopt hijab. The responses suggest that most participants are aware that male family members, friends and/or relatives show preference toward women who adopt hijab
13.	The man I love, admire and/or respect prefers women who wear hijab	72% of participants said yes	
14.	If I stop wearing hijab, others would probably talk negatively behind my back	76% of participants said yes	Questions no. 13, 14 and 15 are probing the possible influence of social stigma and sanction to those who choose not to wear hijab. The responses suggest that most participants are aware of the possible consequences of choosing not to wear hijab
15.	If I stop wearing hijab, I would not be approved by others (e.g., families, friends, neighbors, etc.)	34% of participants said yes	
16.	If I stop wearing hijab, I would be condemned by others (e.g., families, friends, neighbors, etc.)	52% of participants said yes	

Lele et al. (2013) define choice as follows:

“The event of choosing one and only one discrete unit where all members of this set are assumed to be **equally accessible** and known to the individual at the time of choice. (p. 1186)

Table 3 suggests that the claim that hijab adoption is a personal choice cannot hold. Instead, the decision to adopt hijab is actively shaped by their environment (e.g., parents and societies). While choosing between red hijab and blue hijab can be a personal choice; however, in certain communities, wearing or not wearing hijab in public may not be a personal choice as many Muslim women claimed it was. That is because most participants were nurtured to adopt hijab since childhood and were grown up in an ecosystem where their close female circles also wore hijab. Moreover, most participants were fully aware that there were social stigma and sanctions for choosing not to wear hijab hence in the beginning this option (to not wear hijab) is not equally accessible to them. However, I do not deny the possibility that there are Muslim women who embrace hijab as a personal choice. A study by Hussein (2007) provides an interesting point of view on the force/choice discourse. She argues that *“Muslim women ‘negotiate’ rather than ‘choose’ over issues such as dress, and they do not negotiate on equal terms.” (p. 2)*

## Discussion

Muslim women who live in the western countries have to constantly negotiate between secular values and religious values. On the one hand, they must satisfy the expectations from their families to observe Islamic practices (e.g., wearing hijab) and on the other hand, they must also follow the norms in the secular society, such as to refrain from ostentatious display of religiosity (Wayland 1997; Gies 2006). Hair covering in secular societies can be a polemic, wherein Muslim women who adopt such practice are seen as backward and oppressed hence are in need of liberation. This study argues that hijab dissociation is driven by five antecedents: 1) Religious Discrepancy, 2) Low Self-Esteem, 3) Powerlessness, 4) Alienation and 5) Trauma.

There is a link between religiosity and compensatory consumption (Karanika and Hogg 2016; Syahrivar and Pratiwi 2018). The decision to wear or not to wear hijab is deeply rooted in one’s religiosity. Those who are lacking in religiosity will be less inclined to ostentatiously display their religious identities through religious products, such as hijab (Fani et al. 2020). This can also be seen as a mechanism by which people attempt to harmonize between their attitudes about their religions and their actions. Those who disbelieve will eventually manifest their attitudes by dissociating themselves from religious products. The informant of this study frequently asserted that she was a non-believer.

Second, lack of self-esteem has been revealed as antecedent of compensatory consumption (Woodruffe 1997; Koles et al. 2018). People who lack self-esteem will attempt to fix it through consumption activities that enhance their self-worth and confidence. However, it is also possible that people enhance their self-esteem by dissociating themselves from products which caused them to feel inferior. The informant of this study stated how she slowly regained her self-confidence after dissociating herself from hijab.

Third, powerlessness has been linked to compensatory consumption (Rucker & Galinsky 2008). Consumption activities can be geared toward regaining life control or perceived loss of freedom. In the context of dissociation, people stop consuming certain products because they are perceived to be (symbolically) antithesis to freedom, power and control. The informant of this study believed that she had regained her freedom and life control by dissociating herself from hijab.

Fourth, feelings of alienation in general can trigger compensatory consumption (Valence and Fortier 1988; Karanika and Hogg 2016). If consumption activities are seen as means of social integration, I argue in this study that people dissociate themselves from products that could cause the feeling of alienation. In other words, products are preferred or less preferred depending on whether they can improve or weaken one’s social ties with others. The informant of this study explained that her decision to dissociate herself from Islamic identities was partly to avoid discriminations while at the same time getting closer to her chosen networks.

Lastly, trauma due to mental and physical abuse can trigger compensatory consumption. A study by FitzPatrick et al. (2019) centered on victims of domestic violence highlights the role of compensatory consumption in psychological trauma. Among the compensatory mechanisms to reduce psychological trauma is to dissociate oneself from a negative identity as well as the products related to that identity. The informant of this study explained that hijab was seen negatively by her networks and a cause of her discriminations. Thus, she attempted to improve her image by not wearing a hijab.

Following Koles’s argument (2018), I also feel the need to draw the line where dissociation can be compensatory versus compromise (Table 4).

**Table 4:** Dissociation as compensatory versus compromise

	Voluntarily (Internal)	Involuntarily (External)
Compensatory	Religious Discrepancy Low self-esteem	Trauma Alienation Powerlessness
Compromise	Social integration	Product scarcity

In Table 4, I argue that dissociation as a result of social integration (to get closer with the new community) and product scarcity (e.g., as a result of living in non-Muslim-majority countries) are not compensatory consumption but a form of compromise. In this regard, this study expands the literature in compensatory consumption as well as dissociation in particular.

The results of this study support the findings by Finn et al. (2018) who concluded that Canadian Arab youth attempted to mitigate discrimination by dissociating from their Arab identity and by extension, Islamic identity. Also this study supports the findings by White et al. (2012) who concluded that the dissociative response was driven by self-worth concerns and that when social identity was threatened, those higher in independence demonstrated a dissociative response to identity-linked products.

The present study has a limitation. This study is mainly based on the testimonies of one informant who labels herself as an ex-Muslim. As noted by Groenewald (2004), the typical issue with phenomenological study is that it is hard to generalize the results since the reality of a phenomenon is perceived as unique and true to the participant or the informant. Therefore, the focus is on how well the phenomenon can be qualitatively explored and explained irrelevant to the numbers of the participants. A study by Weber (2016) on Muslim digital feminism is an example of a qualitative study revolves around one informant yet is deemed appropriate to unravel the phenomenon under investigation. In this effort, I have incorporated various modes, such as video call, email exchange (essay), online posting, academic articles and a survey involving 25 other participants to improve the validity.

Although it may seem natural at first that the informant dissociated herself from hijab due to her lack of religiosity, the in-depth interviews reveal socio- and psychological factors that contribute to her decisions, such as low self-esteem, powerlessness, alienation and psychological trauma. In this regard, the results of this study expand our understanding on hijab dissociation beyond the scope of religiosity (or lack thereof) as proposed by Fani et al. (2020). At the same time, the results support Ren et al. (2019) who argue that inability to cope with stress and trauma through religious means could induce dissociative response to it.

## Conclusion

I conclude that hijab dissociation is a form of compensatory mechanism, partly in effort to minimize (religious) self-discrepancy, restore self-esteem, gain more personal control, reduce perceived alienation and cope with psychological trauma. However, it is uncertain if the aforementioned objectives are achieved at the end of the day through hijab dissociation since the participant is pretty much still suffering from depression and anxiety. It is also hinted in Stolovy et al. (2015) that dissociation can cause distress. Meanwhile, Koles et al. (2018) argue that compensatory consumption in the end achieved at best a short-term satisfaction that is people who engage in compensatory consumption are unlikely satisfied in the long run because they failed to obtain what they really need in order to solve their sociopsychological issues; hence, compensatory consumption would likely form an infinite loop. In this regard, I can assume that the informant yearns for parental love and affection. By dissociating herself from hijab, not only she attempted to cope with her psychological trauma, she also compensated and obtained love and affection from her like-minded and non-Muslim networks who continuously supported her decision to unveil. This is quite evident in her twitter account where she garners many “loves” for her online postings about unveiling.

In this study, it has been suggested that hijab can be an object that induces an unpleasant memory or a traumatic experience. Hijab as an object can be symbolic because it is linked to event-specific memories, especially concerning one’s interactions with significant others in the past (Zittoun 2009). Since the informant exhibited concerns with her natural skin tone or color and her physical attractiveness in general, it merits an investigation in the future whether hijab colors can reduce the negative feelings. Previous studies indicate that color has the power to regulate and improve mood (Yildirim et al. 2011; Sokolova et al. 2015). Therefore, future studies in religion and health should investigate not only the symbolic meanings of hijab but also how colors can affect these meanings and by extension, the well-being of Muslim women.

I can offer several managerial implications of this study: First, marketing practitioners in the hijab industry should make explicit in their messages on the connection between hijab adoption and their sociopsychological benefits (e.g., improved parent–child relationship). Second, hijab in the western countries needs to be continually redesigned so that beauty and modesty goals can intersect. One objection to the adoption of hijab in the western countries is that it looks conspicuous. Muslim women who observe hijab can be easily spot on in an environment where most women do not wear hijab. Therefore, a form of hijab that is practical but less conspicuous could also be developed. Third, policymakers should develop health programs for Muslim women as they constantly face dilemmas: satisfying the expectations of their families on the one hand and satisfying the expectations of the secular society on the other hand. Moreover, non-Muslim-majority countries may develop self-development programs (e.g., culture and language programs) targeted at Muslim immigrants in order to help them integrate faster with the locals.
